# Postmenopausal Hyperandrogenism due to Ovarian Hyperthecosis

**DOI:** 10.1155/2023/2783464

**Published:** 2023-01-27

**Authors:** Laryssa Santos Metzker, Luyanne Azevedo Cabral Ferreira, Julia Caroliny Nogueira Borges, Mariana Furieri Guzzo, Rodrigo Neves Ferreira, Lucas Luciano Rocha Silva, Rodrigo Monico Cavedo, Antonio Chambô Filho

**Affiliations:** Santa Casa de Misericórdia de Vitória Hospital, R. Dr. João dos Santos Neves 143, Vila Rubim, Vitória, Espírito Santo 29025-023, Brazil

## Abstract

Ovarian hyperthecosis or ovarian stromal hyperplasia is a non-neoplastic functional disorder resulting from the presence of luteinized thecal cells within a hyperplastic ovarian stroma. The condition is more common in postmenopausal women than in those of reproductive age and leads to substantial clinical and laboratory alterations, principally androgenetic alopecia, progressive hirsutism, and elevated testosterone levels. Investigation should include clinical evaluation, laboratory tests, and imaging tests to differentiate between the principal diagnostic hypotheses. The gold standard for diagnosis is histopathology of the ovarian tissue. The present case report describes a woman being followed up as an outpatient at the Santa Casa de Misericórdia Hospital in Vitória, Brazil. The objective in publishing this case report is to add to available data on ovarian hyperthecosis, thus contributing towards improving timely diagnosis and treatment. Early diagnosis and treatment would ensure better quality of life for patients with this condition and better physical and mental health. Moreover, these data should be useful both for the medical community and for future research into this disease.

## 1. Introduction

Androgen levels in the female body decrease significantly following menopause; therefore, the onset of signs and symptoms of hyperandrogenism during this stage of life must be thoroughly evaluated. An initial investigation should be conducted to rule out adrenal tumors and androgen-secreting ovarian tumors. Possible benign causes encompass the use/abuse of anabolic steroids, or cases of Cushing's syndrome and ovarian stromal hyperthecosis [1]. The diagnostic workup involves a clinical evaluation, laboratory tests, and imaging tests to investigate for the principal causes. The laboratory tests include measuring testosterone, dehydroepiandrosterone sulfate (DHEA-S), and 17-hydroxyprogesterone levels. Studies have shown that the first-line option in imaging tests should be transvaginal color Doppler ultrasonography. Nevertheless, an undetected ovarian tumor on Doppler tissue imaging does not completely rule out the possibility of a local lesion since some tumors are very small in size. In such cases, pelvic magnetic resonance imaging is required [2, 3].

Ovarian stromal hyperplasia or hyperthecosis is a functional, non-neoplastic disorder that is more common in postmenopausal woman than in women of reproductive age. However, although severe cases can also occur, they are even less common in young patients [4]. The condition results in significant clinical and laboratory alterations, the principal of which include androgenetic alopecia, progressive hirsutism, and elevated testosterone levels [3, 5]. Other clinical signs of increased androgen levels in women are hirsutism, acne, virilization, anabolic appearance/increased muscle mass, deepening of the voice, obesity, hypertension, and insulin resistance [6].

The gold standard diagnostic test consists of histological investigation performed following bilateral salpingo-oophorectomy [7]. Due to the sparseness of cases of hyperandrogenism in postmenopausal women in the literature, data on any cases of this disease are valuable since they will contribute towards enabling early diagnosis and timely treatment of the disease, thus improving the physical and psychological well-being of affected patients.

This case report describes a woman currently being monitored as an outpatient at the Santa Casa de Misericórdia Hospital in Vitória, Brazil. The report was submitted to the internal review board of the *Escola Superior de Ciências* of the *Santa Casa de Misericórdia de Vitória* and approved under reference number 5.257.649. Written informed consent for publication was obtained from the patient.

## 2. Case Presentation

**Figure 1:**
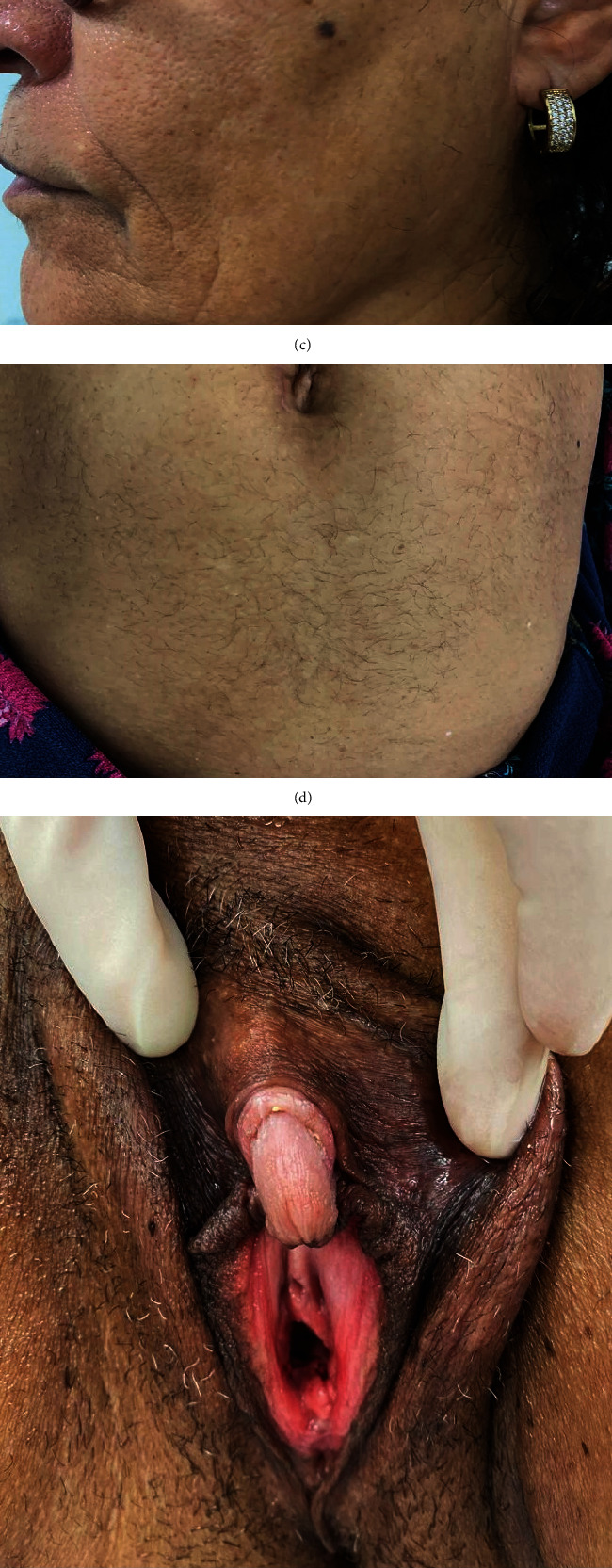
(a) Androgenetic alopecia. (b) Hirsutism (recent depilation). (c) Hirsutism and grade 1 acne on the face. (d) Hirsutism on the abdomen (approximately 4 points on the Ferriman–Gallwey scale). (e) Clitoromegaly and hirsutism on the vulvar region (recent depilation). (f) Male pattern hair growth on the lower limbs.

The patient is a 64-year-old woman who has had three pregnancies. She has a medical history of hypothyroidism and osteoarthritis and became menopausal at 59 years of age. She had central obesity (body mass index of 30 kg/m^2^ and waist circumference of 97 cm), impaired fasting glucose (fasting plasma glucose of 100 mg/dL), and hypercholesterolemia—all signs of metabolic syndrome that are common in patients with this ovarian hyperthecosis. She was being monitored as an outpatient at the Endocrinology Department of a philanthropic hospital in Vitória, Espírito Santo, Brazil, and was referred to the Gynecology Department of the same hospital for further investigation due to a lesion on her ovary detected at transvaginal ultrasonography. In addition, the patient complained of alopecia, hirsutism, and clitoromegaly (Figure 1), as well as deepening of the voice, increased muscle mass, and muscle pain. The androgenic signs and symptoms had been present over the preceding ten years, becoming clearly worse over the past two years. She denied use of exogenous androgens.

**Figure 2:**
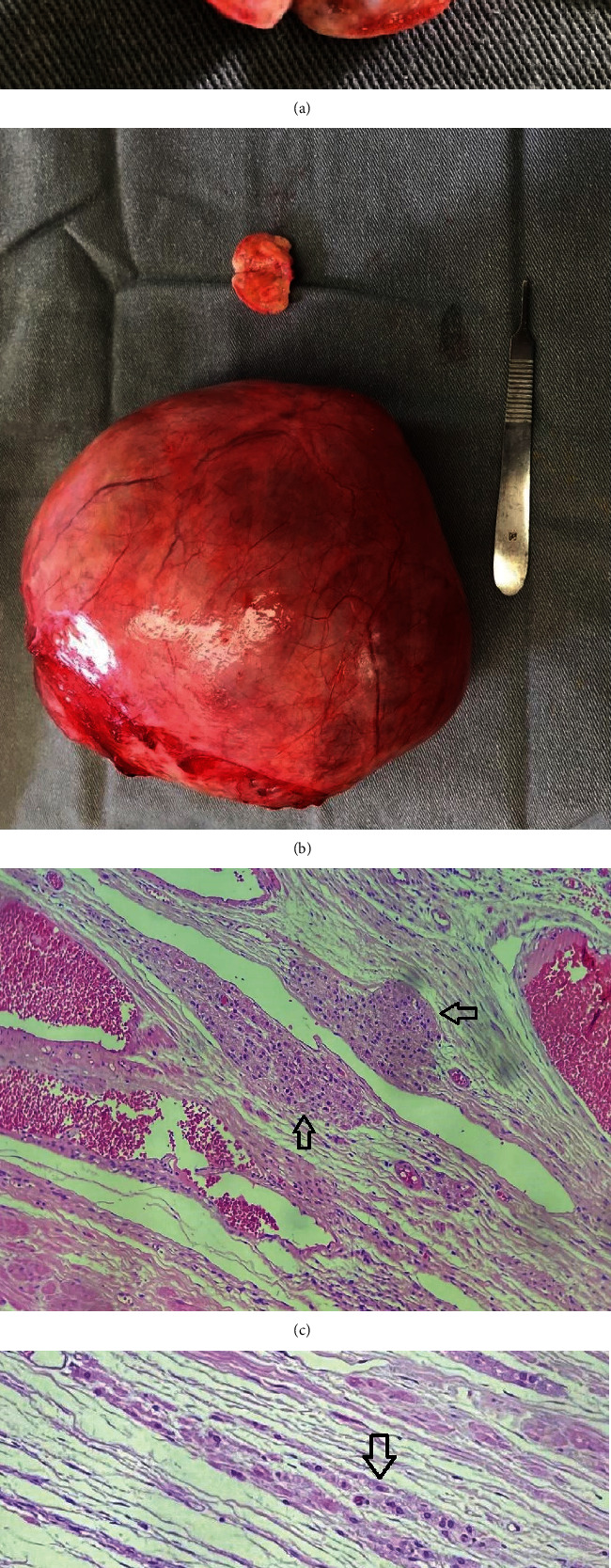
(a) Ovarian adenofibroma on the right ovary. (b) Cystic swelling on the left ovary: serous cystadenofibroma. (c) Clusters of luteinized cells (arrows) on the ovarian stroma; hematoxylin–eosin 400×. (d) Cluster of luteinized stromal cells (arrow) and adjacent stromal edema.

Transvaginal ultrasonography revealed a large cystic mass on the left side of the pelvic cavity with regular borders and a volume of 1,230 mL, possibly of ovarian origin. In addition, there was an irregular hyperechoic area in the central region of the right ovary showing spots with acoustic shadows, possibly corresponding to a calcium deposit, and measuring 3 mm × 3.5 mm × 3 mm. Hormone measurements were requested to investigate further and clarify diagnosis. The results of these tests which are shown in Table 1 revealed severe biochemical hyperandrogenism (serum testosterone: 483.4 ng/dL) with normal DHEAS levels (35.3 *μ*g/dL) for patient's age. A further transvaginal ultrasonography was performed, with results showing the right ovary with normal volume and posterior acoustic shadowing (probably calcium deposit), whereas an image of an anechoic cyst with a volume of 1,214 mL was found on the left ovary. The diagnostic hypothesis reached was that of a Leydig cell tumor, and the patient was sent for exploratory laparotomy to remove the ovaries and fallopian tubes. Ascitic fluid was collected. At macroscopic examination of the surgical specimen, the right ovary appeared normal, with an ovarian cyst being found on the left ovary (Figure 2).

At histopathology, the cystic formation on the left ovary was diagnosed as a serous cystadenoma, with clusters of luteinized cells in the ovarian stroma being compressed by the cyst. The cystic mass weighed 1,513 g, thus confirming the volume found at ultrasonography performed prior to surgery. The luteinized cells had round nuclei and abundant eosinophilic cytoplasm, corresponding to stromal hyperplasia with hyperthecosis. An adenofibroma of 1.1 cm was found on the right ovary (Figure 2). Cytology of the ascitic fluid showed no evidence of neoplastic cells.

One month after surgery, serum testosterone levels dramatically reduced to 13.4 ng/dL, which are shown in Table 2. Concomitantly, imaging tests showed no abnormalities in the pelvis or any suprarenal abnormalities or tumors.

The patient is currently being monitored as an outpatient at the menopause clinic (endocrinology and gynecological endocrinology), and there has been a clear and significant improvement in her laboratory test results, as well as a slight clinical improvement as far as the alopecia is concerned. Some androgenic signs, such as hirsutism and clitoromegaly, still persist. During a consultation, the patient expressed her desire to undergo clitoroplasty and was consequently referred to the vulva outpatient clinic where she is currently undergoing presurgical exams.

## 3. Discussion

Hyperthecosis is a rare disease mostly reported in postmenopausal women, affecting less than 1% of women of reproductive age. In the study conducted by Yance, the mean age of the postmenopausal women with hyperthecosis was 50 years, and they had been experiencing symptoms for a mean of 4–5 years. Furthermore, 97% had hirsutism, and a substantial percentage had increased ovarian volume for their age group and/or unilateral nodules/cysts at presurgical ultrasonography. Patients with deepening of the voice and muscle hypertrophy were 8.5 times more likely to have hyperthecosis, whereas those with testosterone levels >312.5 ng/dL were 31.7 times more likely to have an ovarian tumor rather than hyperthecosis alone [5].

Sarfati et al. evaluated 22 women with hyperandrogenism and reported no significant difference between patients with tumors and those with non-tumoral causes of hyperandrogenism insofar as the natural history of the disease or age at menopause was concerned. According to those authors, testosterone levels >140 ng/dL are strongly indicative of the presence of an androgen-secreting tumor rather than a non-tumoral cause [8]. Several authors have reported that the presence of testosterone levels >200 ng/dL in women of any age is predictive of an ovarian tumor [9, 10]. Nonetheless, in the case reported here, the patient's testosterone level was as high as 483 ng/dL, and no malignant tumor was found. Some dynamic tests can be of use in evaluation of patients with severe hyperandrogenism, especially to differentiate between tumorous and non-tumorous etiologies, including the low-dose dexamethasone suppression test, and the gonadotropin-releasing hormone agonist suppression test [11]. However, even these tests have their own limitations [11]. An additional method, ovarian and adrenal venous catheterization and sampling, has been tested in a small sample of patients, with the investigators concluding that this procedure should not be performed as routine and that computed tomography of the abdomen and ovarian ultrasonography should be used to detect these tumors. Indeed, the aforementioned invasive procedure would be helpful only in cases in which diagnosis remains uncertain and in centers with experience in such cases [12].

In fact, signs of hyperandrogenism are fairly non-specific in the differential diagnosis between an ovarian tumor and a non-tumorous lesion due to the multifactorial origin of the symptoms involved [8, 10]. While hyperthecosis could possibly represent aggravation of polycystic ovary syndrome in the menopause [9], treatment is important in such cases to reduce the consequent malignant transformation of hormone-dependent pathologies [13].

Cumulative data from patients with this disease, including those described in similar case reports, mostly show a significant improvement in hirsutism, partial improvement in frequency of voice, and a very slight improvement or no significant improvement at all in alopecia and hypertrophy of the clitoris following treatment. Therefore, further studies are required to improve early diagnosis and implement timely treatment of the disease, thus minimizing the consequences of hyperandrogenism [5, 13, 14].

In the case reported here, the fact that testosterone levels were as high as 483 ng/dL while no tumor was diagnosed was noteworthy and may be due to the serous cystadenoma found concomitantly with stromal hyperplasia. The mechanical action could have resulted in increased hormone production as mass effect. In the absence of more substantial studies, the hypothesis that hyperthecosis could represent aggravation of polycystic ovary syndrome in the menopause cannot be ruled out [15].

Finally, in the comparative analysis between the patient's pelvic ultrasonography image and that reported in the study conducted by Rousset et al. [16], there is similarity insofar as the ovarian abnormalities are concerned. The ovary of the patient in the present study was increased in size and there was a heterogenic stromal pattern. This supports the findings reported by Rousset et al. in that these changes are able to confirm the diagnosis of ovarian hyperthecosis and rule out the possibility of an androgen-secreting tumor.

## 4. Conclusion

To conclude, in this report, we describe a rare case of severe postmenopausal hyperandrogenism due to ovarian hyperthecosis with significant biochemical resolution following bilateral salpingo-oophorectomy. Cases such as this are valuable when taking into consideration the various different clinical presentations, phenotypes, and variants involved in this disease; therefore, further studies are required to clarify these data. Furthermore, data on such cases would result in earlier diagnosis and treatment, thus improving the quality of life and minimizing complications in patients affected by this disease.

## Figures and Tables

**Table 1:** Initial laboratory tests conducted in a 64-year-old woman with signs of hyperandrogenism.

Hormonal parameters	Serum levels detected	Reference values
Total testosterone (ng/dL)	483.42	9–130
DHEA-S (*μ*g/dL)	35.30	29.7–182.2
FSH (mIU/mL)	80.56	>25.8
SHBG (nmol/L)	26.30	11.7–155.2
Prolactin (ng/mL)	6.15	1.80–20.30
LH (mIU/mL)	35.71	19–54

DHEA-S: dehydroepiandrosterone sulfate; FSH: follicle-stimulating hormone; SHBG: sex hormone binding globulin; LH: luteinizing hormone.

**Table 2:** Laboratory tests prior to and following surgery in a 64-year-old woman diagnosed with ovarian hyperthecosis.

Hormonal parameters	Before surgery	One month after surgery	Seven months after surgery	Reference values
Total testosterone (ng/dL)	483.42	13.77	9.54	9–130
DHEA-S (*μ*g/dL)	35.30	36.50	36.40	29.7–182.2
SHBG (nmol/L)	43.41	26.30	—	11.7–155.2
FSH (mIU/mL)	80.56	72.52	66.00	Superior 25.8
LH (mIU/mL)	35.71	27.21	26.70	19–54
Prolactin (ng/mL)	6.29	—	9.50	1.80–20.30
17-*α* hydroxyprogesterone (ng/mL)	1.61	0.37	0.17	0.13–0.51

DHEA-S: dehydroepiandrosterone sulfate; SHBG: sex hormone binding globulin; FSH: follicle-stimulating hormone; LH: luteinizing hormone. —: Not available on the patient's charts (either not measured or sample lost).

## Data Availability

Data supporting this research article are available from the corresponding author or first author on reasonable request.
